# Functional composition and diversity of leaf traits in subalpine versus alpine vegetation in the Apennines

**DOI:** 10.1093/aobpla/plaa004

**Published:** 2020-03-26

**Authors:** Angela Stanisci, Alessandro Bricca, Valentina Calabrese, Maurizio Cutini, Harald Pauli, Klaus Steinbauer, Maria Laura Carranza

**Affiliations:** 1 EnvixLab, Department of Bioscience and Territory, University of Molise, Termoli, Italy; 2 Department of Science, University of RomaTre, Roma, Italy; 3 Austrian Academy of Sciences, Institute for Interdisciplinary Mountain Research & University of Natural Resources and Life Sciences Vienna, Department of Integrative Biology and Biodiversity Research, Silbergasse, Vienna, Austria

**Keywords:** Calcareous grassland, community-weighted mean traits (CWM_*t*_), leaf dry matter content (LDMC), Mediterranean mountains, plant maximum height (PMH), Rao’s functional diversity (FD_*t*_), specific leaf area (SLA)

## Abstract

Mediterranean high mountain grasslands are shaped by climatic stress and understanding their functional adaptations can contribute to better understanding ecosystems’ response to global change. The present work analyses the plant functional traits of high-elevation grasslands growing in Mediterranean limestone mountains to explore, at the community level, the presence of different plant strategies for resource use (conservative vs. acquisitive) and functional diversity syndromes (convergent or divergent). Thus, we compared the functional composition and diversity of the above-ground traits related to resource acquisition strategies of subalpine and alpine calcareous grasslands in the central Apennines, a mountain region characterized by a dry-summer Mediterranean climate. We used georeferenced vegetation plots and field-measured plant functional traits (plant maximum height, specific leaf area and leaf dry matter content) for the dominant species of two characteristic vegetation types: the subalpine *Sesleria juncifolia* community and the alpine *Silene acaulis* community. Both communities are of particular conservation concern and are rich in endemic species for which plant functional traits are measured here for the first time. We analysed the functional composition and diversity using the community-weighted mean trait index and the functional diversity using Rao’s function, and we assessed how much the observed pattern deviated from a random distribution by calculating the respective standardized effect sizes. The results highlighted that an acquisitive resource use strategy and relatively higher functional diversity of leaf traits prevail in the alpine *S. acaulis* community, optimizing a rapid carbon gain, which would help overcome the constraints exerted by the short growing season. The divergent functional strategy underlines the co-occurrence of different leaf traits in the alpine grasslands, which shows good adaptation to a microhabitat-rich environment. Conversely, in the subalpine *S. juncifolia* grassland, a conservative resource use strategy and relatively lower functional diversity of the leaf traits are likely related to a high level resistance to aridity over a longer growing season. Our outcomes indicate the preadaptation strategy of the subalpine *S. juncifolia* grassland to shift upwards to the alpine zone that will become warmer and drier as a result of anthropogenic climate change.

## Teaser

Mediterranean high mountain grasslands are shaped by climatic stress and understanding their functional adaptations can contribute to better understanding ecosystems’ response to global change. We analysed, at the community level, above-ground plant strategies for resource use and functional diversity syndrome in calcareous grasslands. We observed a more acquisitive resource use strategy and a higher functional diversity in the alpine with respect to the subalpine grasslands in which instead plant traits look more conservative. Subalpine grasses seem more resistant to aridity and well preadapted for shifting upwards into the alpine zone which is getting warmer and drier.

## Introduction

Understanding functional trait composition and diversity at the community level is one of the central challenges of modern ecology (Grime 2006; Cornwell and Ackerly 2009; Myers-Smith *et al.* 2019). Species traits inform the response of plants to the environment (Lavorel and Garnier 2002), and ecological research addressing functional traits can provide a better understanding of the response of natural ecosystems to ongoing anthropogenic global change (Laliberté and Legendre 2010).

Plant functional traits are measurable features affecting the performance of species in a given environment and are of fundamental relevance to community assembly and dynamics, providing insights into how environmental factors shape biodiversity patterns at continental, regional and local scales (Garnier *et al.* 2004; McGill *et al.* 2006; Violle *et al.* 2007; Chelli *et al.* 2019). Their measurement provides a powerful approach to assess ecosystem transformations in response to changing habitat conditions (Wright *et al.* 2004; Ackerly and Cornwell 2007; Kleyer *et al.* 2008; Suding *et al.* 2008).

Recent ecological research on arctic and alpine tundra has shown that habitat conditions are swiftly dynamic under climate change (Pauli *et al.* 2012; Winkler *et al.* 2016; Bjorkmann *et al.* 2018) and that high mountain vegetation tends to respond with consistent variations in plant species composition and turnover (Myers-Smith *et al.* 2011; Epstein *et al.* 2013; Matteodo *et al.* 2013; Jiménez‐Alfaro *et al.* 2014; Venn *et al.* 2014; Rogora *et al.* 2018; Petriccione and Bricca 2019). Even if species identity provides important information related to ecological and evolutionary aspects, further research is needed to better understand how such floristic changes are related to variations in functional trait composition and diversity. Large-scale studies on the plant functional traits of cold-adapted species and communities have been performed in Arctic ecosystems and in the Alps (Bjorkman *et al.* 2018; Myers-Smith *et al.* 2019), and shifts in plant functional traits have been investigated along elevational or latitudinal gradients (review in Körner 2012). In contrast, Mediterranean high mountains remain relatively understudied mainly because of the large gap in knowledge concerning field-measured trait data and the high proportion of endemics for which dedicated field surveys are needed (Chelli *et al.* 2019).

At the community level, plant height and leaf traits are recognized indicators of ecosystem functioning (Diaz *et al.* 2004; de Bello *et al.* 2010). The spatial pattern of these commonly measured plant traits (e.g. plant maximum height (PMH), specific leaf area (SLA) and leaf dry matter content (LDMC)) often responds to the underlying heterogeneity in environmental conditions (Hodgson *et al.* 2011; Bjorkman *et al.* 2018).

One example of such ‘trait–environment’ relationships can be observed in mountain areas in which slopes at lower elevation and summit vegetation tend to adopt different ecological strategies (Read *et al.* 2014). The former is characterized by mild temperatures and fertile soils and preferentially have acquisitive-trait species (tall-broadleaf herbaceous plants) with relatively higher competitive ability and faster resource acquisition capacity (Raich and Schlesinger 1992). Summit vegetation is characterized by low temperatures, a short growing season and low resource availability that promote the occurrence of stress-tolerant species that invest more carbon on a per-leaf basis and develop a resource-conservative trait syndrome (Körner *et al.* 1989; Körner 2012).

In Mediterranean high mountain vegetation, the functional composition and diversity at the community level have not been investigated to date, and few studies have focused analysing functional traits along an elevational gradient. Among the existing studies, seminal work showed a positive correlation of leaf size and leaf dry matter content with water availability and soil pH (Gutiérrez-Girón and Gavilán 2013). Recently, Pescador *et al.* (2015), analysing mountain grasslands in Spain, detected a significant increase in the functional diversity of SLA and LDMC with increasing elevations. In Mediterranean mountains, in addition to the influence of low temperatures, summer drought also plays a key role in shaping biodiversity. The soil water content on summits tends to be higher than that on slopes at lower elevation, which promotes the occurrence of species ensembles that maximize functional diversity (Pescador *et al.* 2015). On the other hand, a very recent work analysing grasslands along an altitudinal gradient in Italy did not identify any significant trend in functional diversity for leaf traits (Bricca *et al.* 2019).

In consideration of the above, the present work attempts to describe the functional composition and diversity of above-ground traits at the community level in Mediterranean mountain grasslands, investigating which functional plant resource use strategy (conservative or acquisitive) performs better in two typical and widespread plant communities of the Apennines. We focused on the subalpine *Sesleria juncifolia* community growing on calcareous slopes and the alpine *Silene acaulis* community growing on calcareous ridges, and we compared for the first time their functional strategies at the community level based on field-measured traits. Vegetation data were extracted from the VIOLA (high mountain VegetatIOn of centraL Apennines) georeferenced database (Stanisci *et al.* 2016a), and traits of plant species, including several endemics, were measured in the field for the first time.

Based on plant maximum height, specific leaf area and leaf dry matter content, we analysed the main above-ground resource use strategies of the alpine and subalpine communities by using the community-weighted mean trait index (CWM; Garnier *et al.* 2004) and functional trait diversity based on Rao’s quadratic entropy (FD; Botta-Dukàt 2005). In particular, we focused on the following questions: (i) How are the main above-ground plant traits (plant maximum height; specific leaf area; and leaf dry matter content) distributed in the dominant species in high-elevation grasslands growing on calcareous Mediterranean mountains? (ii) Do the leaf traits reveal significant differences in resource use strategies (conservative vs. acquisitive) of alpine and subalpine communities? (iii) Do the species coexisting on alpine and subalpine communities express a convergent or a divergent functional diversity pattern?

As the trait-based approach may help to understand ecosystem functioning and its sensitivity to environmental changes (Matteodo *et al.* 2013), our outcomes may contribute to understanding which plant functional traits and resource use strategy may be favoured in Mediterranean high-elevation ecosystems in a warmer climate.

## Methods

### Study area and vegetation data

We analysed the subalpine *S. juncifolia* community and the alpine *S. acaulis* community, which are widely distributed above the timberline of the main calcareous mountain ranges of the central Apennines (Biondi *et al.* 2014; Evangelista *et al.* 2016; **see****Supporting Information—Fig. S1**). The regional climate at 2200 m in this area is characterized by a summer mean temperature of ~8 °C, precipitation of ~213 mm and a winter mean temperature of ~5 °C below zero (Theurillat *et al.* 2011; Bricca *et al.* 2019). Mean annual temperatures in the analysed area have increased during the last 50 years by 1.7 °C (Evangelista *et al.* 2016; Calabrese *et al.* 2018).

The selected plant communities host a high number of Apennine endemic species, southern European orophytes and Mediterranean montane taxa (Stanisci *et al.* 2005, 2011; Peruzzi *et al.* 2014) and are habitats of European conservation concern (code 6170; European Commission 2013). The *S. juncifolia* community consists of calciphilous stepped and garland grasslands, common in the subalpine zone on rendzina soils, while the *S. acaulis* community consists of patchy grasses growing in wind-scoured fell fields at high elevations on shallow soils rich in debris (Blasi *et al.* 2003).

We analysed 92 georeferenced vegetation plots extracted from the VIOLA database (high mountain VegetatIOn of centraL Apennines; Stanisci *et al.* 2016a; European Vegetation Archive code EU-IT-019; Chytrý *et al.* 2016). The average size of the analysed plots was ~50 m^2^. Fifty-five of these plots are assigned in VIOLA to the subalpine *S. juncifolia* community, and 37 plots are assigned to the alpine *S. acaulis* community. The chosen plots satisfied the following criteria: (i) the presence of a pool of the representative species for each plant community (Biondi *et al.* 2014), (ii) a maximized interplot spatial distance (plots distant at least 200 m) and (iii) a comparable vegetation cover. Concerning plot characteristics, the *S. juncifolia* community presents high species richness (mean plot richness = 20,5), while the *S. acaulis* community hosts a low number of species (mean plot richness = 17), many of which are cryophilous and endemic (Stanisci *et al.* 2011). The elevation of the vegetation plots significantly differed, with the *S. juncifolia* ranging from 1650 to 2510 m a.s.l. and the *S. acaulis* ranging from 2160 to 2910 m a.s.l. **[see****Supporting Information—Fig. S2****]**.

The nomenclature of taxa follows Pignatti (2019).

### Trait data

We measured the plant traits of the dominant species. Specifically, for each plot, we ordered the species by cover values, and beginning from the species with the highest cover, we summed the cover values of the species to reach at least 70 % of the vegetation cover in the sample **[see****Supporting Information—Tables S1****and****S2****]**. The percentage of cover of the dominant species per plot varied across the plots, and we assumed a mean value of ~85 (*S. acaulis* community = 81.4 and *S. juncifolia* community = 86.7).

The set of measured taxa included 38 species and subspecies for which the following plant functional traits were measured: maximum plant height at maturity (PMH), specific leaf area (SLA) and leaf dry matter content (LDMC) (Table 1; Westoby 1998; Wright *et al.* 2004). Plant maximum height and leaf trait data were measured in the field with a representative number of replicates conforming to the standardized protocol proposed by Pérez-Harguindeguy *et al.* (2013). In particular, in the 2016 and 2017 summer seasons, we measured PMH (cm) for at least 10 different individuals of each species and SLA (mm^2^ mg^−1^) and LDMC (g mg^−1^) for at least 10 healthy fully expanded leaves of 10 different individuals of each species, for a total of more than 1600 measurements **[see****Supporting Information—Table S3****]**.

**Table 1.** List of measured plant traits (and acronym), along with their description, the associated plant function and bibliographic references.

Trait	Description	Plant function	References
Plant maximum height (PMH)	The distance between the upper boundary of the main photosynthetic tissues on plant and the ground level (in cm).	Competitive ability, dispersal capacity	Diaz *et al.* (2016); Pérez-Harguindeguy *et al.* (2013)
Specific leaf area (SLA)	The ratio between leaf area (mm^2^) and dry weight (mg).	Resource exploitation and conservation; protection against hazard, photosynthetic capacity	Pérez-Harguindeguy *et al.* (2013); Garnier *et al.* (2001); Shipley *et al.* (2005)
Leaf dry matter content (LDMC)	The ratio between leaf dry weight (mg) and the respective fresh weight (g).	Resource exploitation and conservation; protection against hazard, leaf lifetime	Pérez-Harguindeguy *et al.* (2013); Garnier *et al.* (2001)

Trait measurements were obtained for the first time for most of the species because they were not available in the existing databases (e.g. TRY; Kattge *et al.* 2011). In addition, some species, for which trait measurements already existed, were measured again to collect more reliable site-specific measurements and to reduce the effects of intraspecific trait variability (Puglielli *et al.* 2015; Tardella *et al.* 2017).

The species mean values of the measured individual traits were analysed.

### Data analysis

We first measured the differences in floristic composition among the two plant communities by a similarity analysis using a one-way analysis of similarities (ANOSIM) test (9999 permutations). Analysis of similarities is a non-parametric statistical test that assesses the differences between two or more groups based on a ranked dissimilarity matrix (Clarke 1993). Then, we explored which species contributed most consistently to the observed differences between vegetation types using a similarity percentage procedure (SIMPER—Clarke 1993—software PAST; Hammer *et al.* 2008).

We synthesized and compared the functional trait composition and diversity of the subalpine *S. juncifolia* community and the alpine *S. acaulis* community by using two complementary metrics (Ricotta and Moretti 2011): community-weighted mean trait values (CWM; Garnier *et al.* 2004) and functional trait diversity based on Rao’s quadratic entropy (FD; Botta-Dukàt 2005). CWM_*t*_ was calculated as follows (Garnier *et al.* 2004):

$$\begin{array}{l} CWM_{t}=\underset{i=1}{\overset{n}{\sum }}p_{i}x_{i} \end{array}$$

where CWM_*t*_ is the community-weighted mean value of a given functional trait (*t*), *p*_*i*_ is the relative abundance of the *i*th species, *x*_*i*_ is the mean trait value of species *i*, and *n* is the number of species. We calculated CWM_*t*_ separately for each trait (CWM_PMH_, CWM_SLA_, CWM_LDMC_) and for each plant community.

The functional diversity for each functional trait for each plant community was calculated with Rao’s quadratic entropy (FD; Botta-Dukàt 2005) as follows:

$$\begin{array}{l} FD_{t}=\underset{ij}{\overset{n}{\sum }}d_{ji}p_{i}p_{j} \end{array}$$

where *d*_*ij*_ is the functional distance between species *i* and *j* measured by the Gower distance (Pavoine *et al.* 2009) and *p* is the relative abundances of the *i*th and *j*th species (Rao 1982). The FD_*t*_ index equals the sum of the dissimilarity in the trait space among all possible pairs of species, weighted by the product of the species’ relative abundance. The parameter *d*_*ij*_ varies from 0 (two species have exactly the same trait values) to 1 (two species have completely different trait values).

To quantify how much the observed pattern deviated from a random distribution, we calculated the standardized effect size (SES) for CWM_*t*_ and FD_*t*_ (de Bello 2012; Mason *et al.* 2013) according to the following formula (Gotelli and McCabe 2002):

$$\begin{array}{l} SES=\;\;\;\frac{I_{obs}-I_{sim}}{\sigma_{sim}} \end{array}$$

where *I*_obs_ is the functional observed value, *I*_sim_ is the mean of the functional expected values, and σ is the standard deviation of the functional expected values. Expected values were calculated by shuffling trait values across all species 999 times (Botta‐Dukát and Czucz 2016; Vojtkó *et al.* 2017). This procedure is suitable for detecting both the higher and lower CWM_*t*_ and FD_*t*_ observed values compared to those based on random expectation (Botta‐Dukát and Czucz 2016; Zelený 2018) and helps break the link between trait information and species composition. The analysis of CWM_*t*_ SES allowed us to explore the eventual presence of the assembly rule processes (Vojtkó *et al.* 2017; Bricca *et al.* 2019); for example, for functional diversity (FD_*t*_), SES > 0 indicated observed values higher than expected (‘functional divergence’), while SES < 0 indicated observed values lower than expected (‘functional convergence’) (de Bello 2012). Trait values were log-transformed before the calculation (Májeková *et al.* 2016). Community-weighted means were calculated with the function *functcomp* in the ‘FD’ R package version 1.0 (Laliberté *et al.* 2014). FD_*t*_ was calculated with the *Rao* function proposed by de Bello *et al.* (2010), which also provided the Jost correction (1/1 − FD_*t*_). We assessed the adequateness of the selected percentage cover cut-off for the dominant species by sensitivity analysis as follows: we calculated CWM_*t*_ and FD_*t*_ by gradually reducing the dominant species cover by 5 % starting from the observed cover, and we assessed the correlation of the new index values with original data values (Májeková *et al.* 2016, function *traitor in r*; **see****Supporting Information—Table S2**). We observed a high correlation between indexes calculated with reduced data up to 70 % of the original cover.

Then, we also compared plant communities and tested the presence of significant differences in functional trait composition (CWM_*t*_) and diversity (FD_*t*_) using the Mann–Whitney *U*-test (run with the function wilcox.test in stats R-package), and we graphically represented the significant results using box plots.

## Results

The floristic comparison between the selected subalpine and alpine communities highlighted significant differences that were mainly caused by variations in species abundance, such as 87 % of the analysed species are present in both plant communities **[see****Supporting Information—Table S3****]**. The most frequent species in both plant communities are *Helianthemum oelandicum* subsp. *alpestre*, *Carex kitaibeliana* subsp*. kitaibeliana* and *Anthyllis vulneraria* subsp*. pulchella*. Nevertheless, there is a set of species occurring only in the subalpine grassland: *Brachypodium genuense*, *Cytisus spinescens*, *Carex macrolepis*, *Carex humilis* and *Helianthemum nummularium* subsp*. grandiflorum.* The similarity percentage procedure (SIMPER) analysis showed that the *S. juncifolia* community is characterized by significantly high occurrences of *S. juncifolia* subsp*. juncifolia*, *Globularia meridionalis*, *Anthyllis montana* and *C. humilis.* On the other hand, the *S. acaulis* community is distinguished by significantly high occurrences of *Salix retusa*, *Armeria gracilis* subsp. *majellensis* and *S. acaulis* subsp*. bryoides* (Table 2).

**Table 2.** Plant species contribution (sp contr. %) to the difference between the subalpine *Sesleria juncifolia* community and the alpine *Silene acaulis* community, assessed by the similarity percentage procedure (SIMPER, Clarke 1993). % plots: percent of the plots in which the species occur. *Endemic taxon.

Taxon	sp contr. (%)	*Sesleria juncifolia* community (% plots)	*Silene acaulis* community (% plots)
*Sesleria juncifolia* subsp*. juncifolia*	5.59	94.60	22.20
*Salix retusa*	4.86	7.14	66.70
*Globularia meridionalis*	4.67	60.70	2.78
*Anthyllis montana*	4.66	62.50	8.33
*Armeria gracilis subsp. *majellensis*	4.58	14.30	69.40
*Silene acaulis* subsp. *bryoides*	4.14	26.80	69.40
*Carex humilis*	4.12	57.10	0
*Edraianthus graminifolius* subsp. *graminifolius*	3.96	76.80	50.00
*Helianthemum oelandicum* subsp. *alpestre*	3.70	89.30	58.30
*Pedicularis elegans	3.62	53.60	44.40
*Anthyllis vulneraria* subsp. *pulchella*	3.33	69.60	72.20
*Androsace villosa* subsp. *villosa*	3.29	39.30	36.10
*Trinia dalechampii*	3.23	44.60	19.40
*Carex kitaibeliana* subsp. *kitaibeliana*	3.11	73.20	75.00
*Festuca violacea subsp. *italica*	3.03	23.20	41.70
*Galium magellense	2.98	3.57	38.90
*Thymus praecox* subsp. *polytrichus*	2.80	33.90	22.20
*Helictochloa praetutiana	2.67	30.40	25.00
*Arenaria grandiflora* subsp. *grandiflora*	2.53	8.93	36.10
*Myosotis graui	2.47	10.70	30.60
*Iberis saxatilis* subsp*. saxatilis*	2.28	28.60	16.70
*Bromopsis erecta*	2.27	28.60	2.78
*Kobresia myosuroides*	2.23	5.36	33.30
*Leontopodium nivale*	2.17	26.80	19.40
*Potentilla crantzii* subsp. *crantzii*	2.06	12.50	27.80
*Aster alpinus* subsp. *alpinus*	2.01	30.40	5.56
*Cerastium thomasii	1.93	1.79	25.00
*Oxytropi scampestris* subsp. *campestris*	1.73	23.20	11.10
*Alyssum cuneifolium* subsp*. cuneifolium*	1.40	1.79	19.40
*Valeriana saliunca	1.29	5.36	19.40
*Viola magellensis	1.25	1.79	19.40
*Cerastium tomentosum	1.24	14.30	2.78
*Brachypodium genuense	1.22	16.10	0
*Cytisus spinescens*	0.94	10.70	0
*Potentilla apennina* subsp. *apennina*	0.86	10.70	2.78
*Carex macrolepis*	0.71	8.93	0
*Androsace vitaliana subsp. *praetutiana*	0.57	3.57	5.56
*Helianthemum nummularium* subsp*. grandiflorum*	0.52	7.14	0

Concerning species trait values **[see****Supporting Information—Table S3****]**, the graminoids *B. genuense* and *Bromopsis erecta* had the greatest mean plant maximum height values (36.12 and 32.72 cm, respectively), whereas the alpine small cushion plants, *Androsace vitaliana* subsp*. praetutiana* (0.55 cm), *S. acaulis* subsp*. bryoides* (1.25 cm) and *Androsace villosa* subsp*. villosa* (0.18 cm), showed the lowest values. Moreover, the mean SLA values were particularly high for the endemic alpine forbs *Galium magellense* (23.2 mm^2^ mg^−1^), *A. gracilis* subsp*. majellensis* (19.46 mm^2^ mg^−1^) and *Myosotis graui* (19.24 mm^2^ mg^−1^) and very low in the dwarf shrubs *H. oelandicum* subsp*. alpestre* (8.31 mm^2^ mg^−1^), *G. meridionalis* (8.38 mm^2^ mg^−1^) and *A. montana* (8.94 mm^2^ mg^−1^). The highest mean LDMC values were measured in the graminoids *B. genuense* (501.68 mg g^−1^), *B. erecta* (477.28 mg g^−1^) and *Helictochloa praetutiana* (444.70 mg g^−1^), and the lowest values were measured in the alpine forbs *M. graui* (190.29 mg g^−1^) and *Valeriana saliunca* (204.14 mg g^−1^).

Notably, most of the species with very light leaves (e.g. *M. graui*, *V. saliunca*, *Viola magellensis* and *A. gracilis* subsp*. majellensis*) were endemic alpine forbs.

In general, species with low PMH, high SLA and low LDMC were more frequent in the high-elevation alpine community, whereas in the subalpine grassland, tall herbaceous species with heavier leaves and lower SLA were dominant.

**Figure 1.**
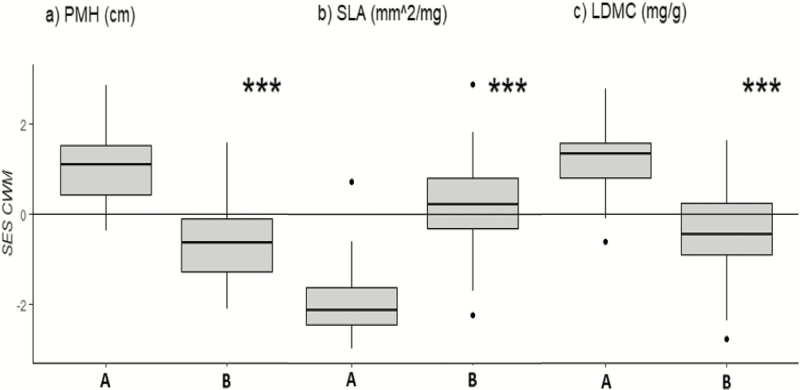
Box plots comparing the *standardized effect size* (SES) CWM_*t*_ values of subalpine *Sesleria juncifolia* (A on the horizontal axis) and alpine *Silene acaulis* (B on the horizontal axis) communities. The differences in the CWM_*t*_ are significant for (A) plant maximum height (PMH), log cm; (B) specific leaf area (SLA), log mm^2^ mg^−1^; and (C) leaf dry matter content (LDMC), log mg g^−1^. Statistical significance according to the Mann–Whitney *U*-test is represented by asterisks (non significant *P* > 0.05; *P < 0.05; **P < 0.01; ***P < 0.001).

**Figure 2.**
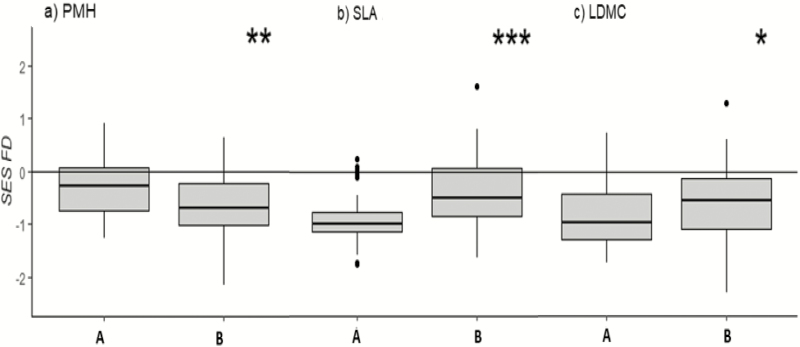
Box plots comparing the *standardized effect size* (SES) FD_*t*_ values of subalpine *Sesleria juncifolia* (A on the horizontal axis) and alpine *Silene acaulis* (B on the horizontal axis) communities. The differences in the FD_*t*_ are significant for (A) plant maximum height (PMH), log cm; (B) specific leaf area (SLA), log *t* mm^2^ mg^−1^; and (C) leaf dry matter content (LDMC), log mg g^−1^. Statistical significance according to the Mann–Whitney *U*-test is represented by asterisks (non significant *P* > 0.05; *P < 0.05; **P < 0.01; ***P < 0.001).

The analysis of functional trait composition (CWM_*t*_) and diversity (FD_*t*_) revealed significant differences between the two plant communities for all the considered traits: PMH (CWM_PMH_, FD_PMH_), LDMC (CWM_LDMC_) and SLA (CWM_SLA_, FD_SLA_). The alpine *S. acaulis* community was characterized by a lower SES CWM_PMH_, a lower SES CWM_LDMC_ and a higher SES CWM_SLA_ compared to those of the subalpine *S. juncifolia* community (Fig. 1). Moreover, the analysis of functional diversity (FD_*t*_) revealed a higher convergence for PMH (lower SES FD_PMH_) in the alpine *S. acaulis* community than in the *S. juncifolia* community (Fig. 2A). In contrast, in comparison to the *S. acaulis* community, the *S. juncifolia* community was characterized by higher convergence in both leaf traits (lower SES FD_SLA_ and FD_LDMC_) (Fig. 2B and C).

## Discussion

Our analysis provided new insights into the above-ground resource use strategies of alpine and subalpine species growing in the Mediterranean high mountains and highlighted significant differences in the functional composition and diversity between the subalpine *S. juncifolia* community and the alpine *S. acaulis* community. Such communities are changing under climate change (Evangelista *et al.* 2016), and according to our results, they adopt a differentiated trait syndrome. The differences in functional strategies of the compared communities are mainly driven by differences in species cover and secondarily by differences in species composition.

### Community-weighted mean traits

The analysis at the community level of the measured plant traits showed differentiated functional syndromes in the compared vegetation types. In comparison to the *S. acaulis* grasslands, the *S. juncifolia* grasslands growing on slopes at lower elevations showed higher mean values of plant maximum height (CWM_PMH_). A comparable trend was recorded in the subalpine grasslands of the Alps (Körner 2003; Pellissier *et al.* 2010), and it was explained by the longer growing season and the warmer habitat conditions that, at lower elevation in the mountains, favour the occurrence of taller plants (Körner 1989). Furthermore, in such a relatively mild environment, interspecific competition for light becomes a key driver in shaping dense grassland assembly (Venn *et al.* 2014). The low values of CWM_PMH_ in the alpine *S. acaulis* grasslands summarize the well-known trend of plant size reduction at the community level due to the temperature decrease at higher altitudes, which is a feature shared by virtually all mountains (e.g. Körner 1989; Pellissier *et al.* 2010; Dainese *et al.* 2012; Spasojevic and Suding 2012; de Bello *et al.* 2013; Pescador *et al.* 2015). At higher elevations where the temperature is low and the growing season is short, a low-stature growth form is advantageous over taller forms because daytime temperatures near the ground are far higher than those in the free atmosphere. Plants also benefit from the thermostability of soils, as the compact shape allows heat storage, and a relatively lower size provides protection against wind and allows efficient recycling of nutrients and water storage (Körner 2003).

The community-weighted mean indexes for the leaf traits indicated that the alpine *S.* acaulis grassland was characterized by a higher SLA value (CWM_SLA_) and lower LDMC value (CWM_LDMC_), which seems to be opposite of the common view of altitude adaptation by plants to low-temperature and harsh environmental conditions. Indeed, previous studies have found that plants tend to have relatively smaller leaf area and lower water content at higher altitudes in the Alps and other European summits (Kӧrner 1989, 2003; de Bello *et al.* 2013; Rosbakh *et al.* 2015). Harsh environmental conditions and low resource availability on mountain summits have been reported repeatedly in relation to promoting stress-tolerant species that invest more carbon on a per-leaf basis (Kӧrner 2012), resulting in lower SLA values (Kӧrner *et al.* 1989; Pescador *et al.* 2015). Thus, our conflicting finding is surprising but is likely related to a specific strategy that optimizes rapid carbon gain, which would help overcome the constraints exerted by the short growing season (Gonzalo-Turpin and Hazard 2009) coupled with the summer aridity, which characterizes high elevations in the central Apennines (Olano *et al.* 2013). To optimize the carbon gain during a short vegetative period, some plants quickly grow during the pre-reproductive phase and shift the acquired resources to seeds before the end of the growing season (Garnier 1992). In fact, a number of alpine species seem to adopt a ruderal strategy (*sensu*Grime 1979) early in the growing season, when relatively milder temperatures and good soil nutrient conditions prevail and water is amply available. Then, in midsummer, young leaves may already be desiccated (Körner 2003), and plants shift to a stress-tolerant mode to overcome the drought effects caused by low precipitation, especially in shallow calcareous soils with low water retention capacity. In this context, a study in the Swiss Alps demonstrated that the highest summits are dominated by stress-tolerant ruderal species (Matteodo *et al.* 2013), which match a conservative syndrome for some plant traits and an acquisitive syndrome for other traits, at least seasonally.

Interestingly, species with higher SLA and lower LDMC values in the alpine *S. acaulis* community are the endemic species *G. magellense*, *M. graui*, *A. vitaliana* subsp*. praetutiana* and *V. magellensis* that belong to different taxonomic families **[see****Supporting Information—Table S3****]**. Such similarities may represent convergent adaptations to the harsh environmental conditions of the summit areas in these Mediterranean calcareous mountains, leading to similar morphological and physiological traits (Ackerly and Reich 1999).

High mean SLA values in the high-elevation plant communities of the Alps were recorded only in snowbed species (Rosbakh *et al.* 2017). These species exhibit rapid production of horizontal, large, low-cost leaves with a short lifespan and a high SLA in comparison with species growing at early-melting sites (Choler 2005), and this scenario was interpreted as a short phenological phase strategy that assures a relatively higher fitness for the species growing in time-limited habitats.

Regarding the subalpine *S. juncifolia* grassland, the low CWM_SLA_ and high CWM_LDMC_ observed are most likely related to an efficient strategy for nutrient conservation (Westoby 1998; Wright *et al.* 2004). The highest LDMC values were recorded in *Poaceae* and *Cyperaceae***[see****Supporting Information—Table S3****]**, which are relatively more abundant in the low-elevation community. Their presence indicates high biomass accumulation, lateral spread and good competitive capacity (Westoby *et al.* 2002; Cornelissen *et al.* 2003), which are associated with adequate levels of soil organic matter and nitrogen (Myers-Smith *et al.* 2011; Vankoughnett and Grogan 2014). Moreover, leaves with high dry matter content may maintain turgor at a relatively lower water potential and enhance drought tolerance and freezing resistance (Pescador *et al.* 2016). This adaptation to drought and freezing seems to be a general prerequisite of plants in xeric calcareous mountains, such as Mediterranean mountains. The drought effects of the typical dry-summer season in Mediterranean mountains decrease with higher elevations (Giménez-Benavides *et al.* 2007). The constraint arising from limited water availability is most likely more relevant for plant life in subalpine habitats than those in low-temperature habitats (Cavieres *et al.* 2006; Schöb *et al.* 2013). The combined drought and freezing tolerance of grassland species may ensure a type of ‘preadaptation’ to the effects of global warming, as indicated in recent studies concerning species composition and structural changes in central Apennines grasslands over the last four decades (Evangelista *et al.* 2016; Stanisci *et al.* 2016b; Frate *et al.* 2018).

Previous research indicated intraspecific trait variation plays a minor role in high mountain communities’ functional strategies (Pescador *et al.* 2015; Rosbakh *et al.* 2015, Henn *et al.* 2018). Nevertheless, we think that new case studies should also be carried out exploring intraspecific and interspecific variability in a wide variety of taxa and regions to enrich the current ecological knowledge and test whether this general rule is valid in other ecosystems and environmental frames.

### Functional trait diversity

The results of the functional trait diversity analysis showed a convergence of PMH values and a divergence in the SLA and LDMC values in alpine vegetation and an opposite trend for the subalpine grassland. The lower values for FD_PMH_ in the alpine *S. acaulis* community than in the *S. juncifolia* community had a narrow variation range for plant height, which should be related to the constraints exerted by the harsh environmental conditions that characterize the high mountain summits (i.e. abiotic filtering through low-temperature conditions and high wind speed; de Bello *et al.* 2013). In contrast, the higher biotic competition in the dense subalpine grassland probably explains the observed wider variation range for PMH in the subalpine *S. juncifolia* plant communities (Dainese *et al.* 2015).

The divergent functional strategy of leaf traits (SES FD_SLA_ and SES FD_LDMC_) in the alpine *S. acaulis* community indicates the co-occurrence of different leaf traits, allowing improved adaptability in a microhabitat-rich environment (Choler 2005; Stanisci *et al.* 2011) and underlining high diversity in several ecological processes, such as photosynthesis, growth rates, leaf longevity and litter decomposability. Therefore, a relatively higher divergence of SLA values may have positive effects on ecosystem processes such as productivity and nutrient retention in an environmental context where biotic competition is low (Tilman *et al.* 1997).

In contrast, the observed low functional diversity of leaf traits (FD_SLA_ − FD_LDMC_) in the subalpine *S. juncifolia* grassland may reflect a strategy for preventing water loss during the growing season. This functional leaf syndrome should ensure good performance for the species growing in similar drought-stricken habitats (Nunes *et al.* 2017). However, de Bello *et al.* (2012) observed that in arid environments, plant communities can host two sets of functional strategies: tall species with low SLA or short species with high SLA. Our findings are consistent with those from recent studies on Spain (Pescador *et al.* 2015) that showed the presence of high functional diversity in terms of SLA at higher elevation. In contrast, a similar study conducted in the Alps detected the opposite pattern for SLA, finding greater diversity in this trait at low altitudes than at high altitudes (de Bello *et al.* 2013).

The different patterns in leaf functional diversity may be explained by climatic differences between the Alps and Mediterranean high mountains. Indeed, as assessed by Pescador *et al.* (2015), the latter are characterized by the presence of two opposing gradients (cold temperature vs. summer drought) that may cause more stressful conditions in subalpine grasslands that experience greater water limitations during summer, which would result in leaf trait convergence.

## Conclusion

Our outcomes contribute to improving the current knowledge about the functional syndrome at the community level of alpine and subalpine vegetation in Mediterranean mountains, where cold temperatures and summer droughts greatly affect species assemblages and community functional responses.

Moreover, we contribute to reducing the gap in information describing plant traits of high-elevation Apennine endemic species, for which no functional measured data have been obtained to date.

The leaf traits of the alpine *S. acaulis* community at higher elevations with an acquisitive resource use strategy and a leaf trait divergence may have positive effects on ecosystem resilience to moderate global warming effects.

On the other hand, the leaf traits with conservative resource use strategy and leaf trait convergence of the subalpine *S. juncifolia* grassland may plant communities to be more resistant to aridity. This could make them more able to shift upwards on summit slopes that become warmer and drier as a result of global warming.

It would be valuable to conduct similar studies on plant trait patterns in other Mediterranean alpine grasslands, involving multisite comparisons with larger data sets and considering a larger proportion of species in the target communities. This would enhance our understanding of the functional syndrome of these communities and contribute to reducing the gap in knowledge on the functioning of alpine ecosystems of the spatially restricted and highly fragmented Mediterranean high mountains.

## Supporting Information

The following additional information is available in the online version of this article—

**Figure S1.** Localization of the study area and the mountain massifs of central Apennines (Abruzzi Region) where plant traits were collected.

**Figure S2.** Box plots comparing the elevation range of vegetation plots sampled on *Sesleria juncifolia* and *Silene acaulis* communities.

**Table S1.** List of plots (*N*), the attribution to the two compared communities, subalpine *Sesleria juncifolia* community and alpine *Silene acaulis* community (Community), the massif in which plots were collected (Locality), the plot’s geographic coordinates (Coordinates WGS84), the total cover of species per plot (Total cover), the cover of the dominant species for which traits were measured (Dominant species cover) and the cover of dominant species as a percentage of the total cover of species in the plot (Dominant species cover (%)).

**Table S2.** Sensitivity analysis obtained by calculating the correlation among CWM_*t*_ and FD_*t*_ values, considering the overall cover (cover 100 %) and the values that CWM_*t*_ and FD_*t*_ assumed using gradually reduced species cover (step of 5 %).

**Table S3.** List of the dominant species (species, asterisks indicate the Apennine endemic taxa), along with their taxonomic family (Family), Growth form (GF—Raunkiaer 1934; Pignatti 2019; CH FRUT: fruticose chamaephyte, CH PULV: pulvinate chamaephyte, CH REPT: reptant chamaephyte, CH SUFFR: suffruticose chamaephyte, H SCAP: scapose hemicryptophyte, H ROS: hemicryptophyte with rosette, H CAESP: caespitose hemicryptophyte), number of plots in which the species occurs (N pl), mean measured traits (SLA: specific leaf area, LDMC: leaf dry matter content, PMH: maximum plant height), percentage of plots in which the species occurs into the alpine *Silene acaulis* community (% pl alpine) and into the subalpine *Sesleria juncifolia* community (% pl subalpine).

plaa004_suppl_Supplementary-Material

## Sources of Funding

This study was partially supported by Ministero dell’Istruzione, dell’Università e della Ricerca, in the context of NEXTDATA project, and by Österreichischen Akademie der Wissenschaften, in the context of the project MEDIALPS.

## Contributions by the Authors

Conceptualization: A.S., A.B., M.L.C. Project direction: A.S., H.P. Methodology: M.L.C., A.B. Data collection and field work: A.B., V.C., K.S., M.C. Formal analysis: A.B., V.C. Writing – original draft: A.S., A.B., M.L.C., H.P. Writing – editing: A.S., A.B., M.L.C. Supervision: A.S., M.L.C. All co-authors contributed to revisions.

## Conflict of Interest

The authors declare no conflict of interest.
