# Supplementary material for: Functional composition and diversity of leaf traits in subalpine versus alpine vegetation in the Apennines
Source: AoB Plants. 2020 Mar 26;12(2):plaa004. doi: 10.1093/aobpla/plaa004 (PMC7098876; doi:10.1093/aobpla/plaa004)
Supplement: plaa004_suppl_Supplementary-Material [file plaa004_suppl_supplementary-material.pdf]

Functional composition and diversity of leaf traits in subalpine versus alpine vegetation in the Apennines

Stanisci Angela[1], Bricca Alessandro[2], Calabrese Valentina[1], Cutini Maurizio[2], Pauli Harald[3], Steinbauer Klaus[3], Carranza Maria Laura*[1]

SUPPLEMENTARY MATERIAL

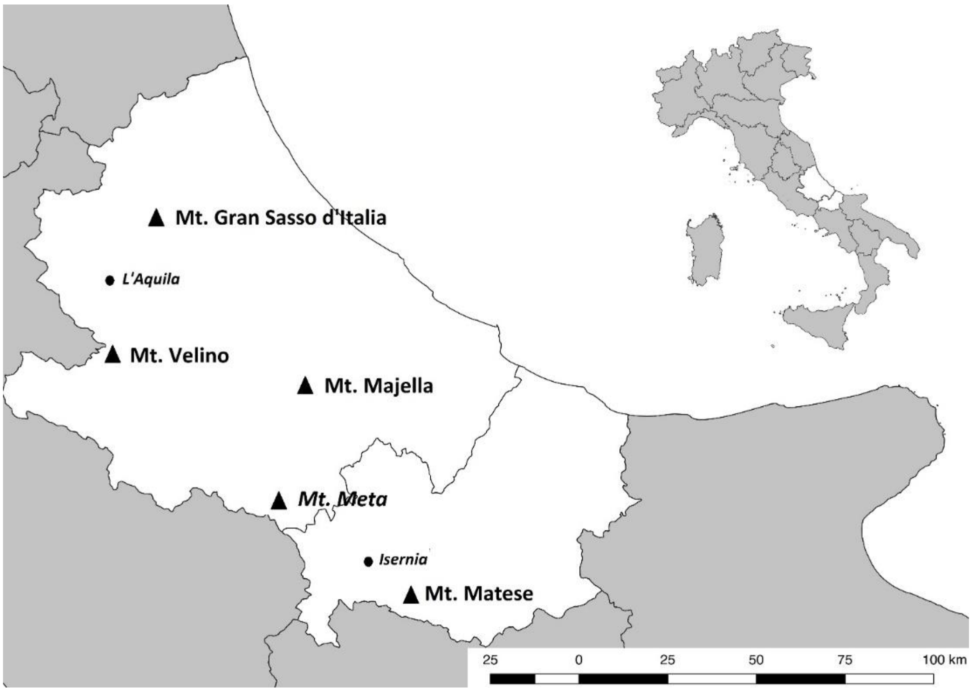

Figure S1 Localization of the study area and the mountain massifs of Central Apennines (Abruzzi Region) where plant traits were collected

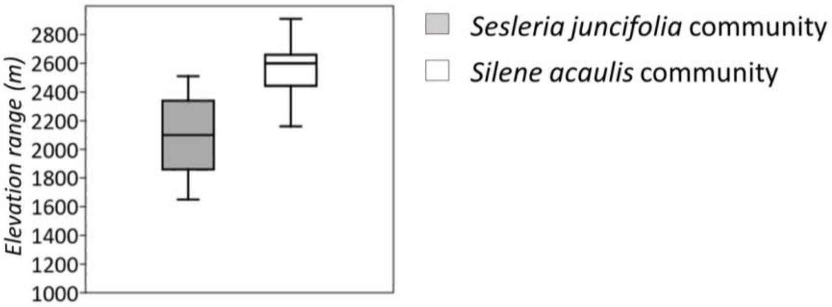

Figure S2 Box-plots comparing the elevation range of vegetation plots sampled on *Sesleria juncifolia* and *Silene acaulis* communities; difference is significant (p<0.001- Mann-Whitney U test, software Past, Hammer et al.2008)

Table S1

List of plots (**N**), the attribution to the two compared communities, subalpine *Sesleria juncifolia* community and alpine *Silene acaulis* community (**Community**), the massif in which plots were collected (**Locality**), the plot's geographic coordinates (**Coordinates WGS84**), the total cover of species per plot (**Total cover**), the cover of the dominant species for which traits were measured (**Dominant species cover**) and the cover of dominant species as a percentage of the total cover of species in the plot **(Dominant species cover (%))**. Notice that the accumulated cover of the species for which traits were measured, expressed as percent of the overall cover of species recorded in the plots, reaches the 85.4% for the subalpine grassland as for the alpine one the 82%.

N	Community	Locality	Coordinates (WGS84)	Total cover	Dominant species cover	Dominant species cover (%)
1	subalpine *Sesleria juncifolia* community	Matese	Long 14.41462559<br>Lat 41.43578272	337.5	240	71.1
2		Matese	Long 14.41666929<br>Lat 41.43237528	412.5	338	81.8
3		Velino	Long 13.40932846<br>Lat 42.13598432	287.5	242	84.0
4		Velino	Long 13.37264535<br>Lat 42.13783955	149.5	107	71.6
5		Velino	Long 13.37709880<br>Lat 42.13365489	250.5	204	81.4
6		Majella	Long 14.09590055<br>Lat 42.0290165	92	87	94.6
7		Majella	Long 14.09078858<br>Lat 42.03145549	124	119	95.6
8		Majella	Long 14.09481499<br>Lat 42.03075312	109	104	95.0
9		Majella	Long 14.09837130<br>Lat 42.02967357	136.5	135	98.5
10		Majella	Long 14.11017599<br>Lat 42.03227914	106	101	95.3
11		Majella	Long 14.09521153<br>Lat 42.03109298	105.5	101	95.3
12		Velino	Long 13.43089695<br>Lat 42.16467665	175	169	96.6
13		Velino	Long 13.34143359<br>Lat 42.17509433	135.5	130	95.9
14		Velino	Long 13.34195214<br>Lat 42.17642806	195	190	97.2
15		Majella	Long 14.10563103<br>Lat 42.03893986	137.5	97	70.2
16		Majella	Long 14.10166563<br>Lat 42.03608535	190	156	82.1

17		Majella	Long 14.10553952<br>Lat 42.03805634	174	140	80.5
18		Gran Sasso	Long 14.08122000<br>Lat 42.11102000	169	152	89.9
19		Majella	Long 14.09089805<br>Lat 42.02788359	62	58	93.5
20		Velino	Long 13.96438724<br>Lat 42.11787763	114.5	109	94.8
21		Velino	Long 13.37414000<br>Lat 42.13426564	97	92	94.3
22		Mainarde	Long 13.94234065<br>Lat 41.68636128	321	303	94.4
23		Gran Sasso	Long 13.73090873<br>Lat 42.42529425	377.5	281	74.4
24		Gran Sasso	Long 13.70076291<br>Lat 42.41720071	263	220	83.5
25		Gran Sasso	Long 13.70064054<br>Lat 42.41328377	263.5	220	83.3
26		Gran Sasso	Long 13.69251145<br>Lat 42.42183188	173	168	96.8
27		Velino	Long 13.37842247<br>Lat 42.18387028	166	124	74.4
28		Velino	Long 13.46466156<br>Lat 42.13891349	171	131	76.6
29		Gran Sasso	Long 13.58187787<br>Lat 42.44518287	283	204	71.9
30		Velino	Long 13.43494150<br>Lat 42.13438826	173	130	74.9
31		Velino	Long 13.41550533<br>Lat 42.13678732	174.5	130	74.2
32		Velino	Long 13.39108390<br>Lat 42.17669032	190	147	77.1
33		Gran Sasso	Long 13.55026000<br>Lat 42.44594000	146.5	141	95.9
34		Velino	Long 13.39506065<br>Lat 42.13730756	213	168	78.6
35		Gran Sasso	Long 14.08676000<br>Lat 42.10070000	185	168	90.5
36		Velino	Long 13.36425959<br>Lat 42.15394921	222	213	95.7
37		Velino	Long 13.42989357<br>Lat 42.16479661	153	152	99.0
38		Velino	Long 13.43115553<br>Lat 42.15863677	115	109	94.3
39		Velino	Long 13.43142838<br>Lat 42.16372589	173	131	75.7

40		Velino	Long 13.37838230<br>Lat 42.18075332	225	183	81.1
41		Gran Sasso	Long 13.62735741<br>Lat 42.44204077	258	204	78.9
42		Gran Sasso	Long 13.57870782<br>Lat 42.46167945	209	169	80.9
43		Gran Sasso	Long 13.64691318<br>Lat 42.45569526	224.5	184	82.0
44		Velino	Long 13.38002135<br>Lat 42.14713536	194.5	191	97.9
45		Velino	Long 13.37922827<br>Lat 42.14426310	318.5	280	87.8
46		Velino	Long 13.46544401<br>Lat 42.13857875	206.5	167	80.9
47		Majella	Long 14.10438041<br>Lat 42.03676501	179.5	162	90.3
48		Majella	Long 14.09158557<br>Lat 42.09074543	276	258	93.5
49		Gran Sasso	Long 13.55993500<br>Lat 42.45675422	121	117	96.3
50		Gran Sasso	Long 13.54297958<br>Lat 42.44687598	234	190	81.0
51		Majella	Long 14.10553952<br>Lat 42.03681032	182	150	82.1
52		Gran Sasso	Long 13.67169772<br>Lat 42.45094021	155	115	74.2
53		Velino	Long 13.45950828<br>Lat 41.80774612	186.5	182	97.3
54		Gran Sasso	Long 13.72071643<br>Lat 42.44044778	124	123	98.8
55		Velino	Long 13.35124032<br>Lat 42.17693667	89	85	95.5
56	alpine *Silene acaulis* community	Majella	Long 14.08052782<br>Lat 42.09347432	157.5	117	74.3
57		Gran Sasso	Long 13.52906698<br>Lat 42.44747482	38	38	98.7
58		Majella	Long 14.09646215<br>Lat 42.05555795	155.5	138	88.4
59		Majella	Long 14.09538120<br>Lat 42.05722066	123	117	94.7
60		Majella	Long 14.09074753<br>Lat 42.09042829	156	151	96.5
61		Gran Sasso	Long 13.55241362<br>Lat 42.44917825	182	178	97.8
62		Gran Sasso	Long 13.55238000<br>Lat 42.44921000	174	155	89.1

63	Gran Sasso	Long 13.56173579<br>Lat 42.47362362	78	76	96.8
64	Majella	Long 14.08367896<br>Lat 42.08913374	87.5	83	94.9
65	Gran Sasso	Long 13.58956308<br>Lat 42.42568849	197	142	71.8
66	Majella	Long 14.09488000<br>Lat 42.08807000	280.5	205	72.9
67	Gran Sasso	Long 13.55885200<br>Lat 42.46952123	231.5	201	86.6
68	Gran Sasso	Long 13.56023749<br>Lat 42.47073444	231.5	200	86.4
69	Gran Sasso	Long 13.55736314<br>Lat 42.46964599	164.5	126	76.6
70	Gran Sasso	Long 13.56583421<br>Lat 42.46939206	164.5	164	99.4
71	Gran Sasso	Long 13.56470631<br>Lat 42.46614502	165	126	76.1
72	Gran Sasso	Long 13.56337533<br>Lat 42.46534736	164.5	125	76.0
73	Majella	Long 14.10954095<br>Lat 42.09416882	198.5	181	90.9
74	Majella	Long 14.10871737<br>Lat 42.09391984	218	164	75.2
75	Majella	Long 14.10880887<br>Lat 42.09335397	196.5	142	72.3
76	Majella	Long 14.10640000<br>42.09885000	363.5	273	75.0
77	Majella	Long 14.09884000<br>42.05726000	633	481	75.9
78	Majella	Long 14.10651000<br>Lat 42.05532000	646.5	533	82.4
79	Majella	Long 14.12273000<br>Lat 42.06890000	631.5	481	76.1
80	Majella	Long 14.11420792<br>Lat 42.10559827	315.5	246	77.8
81	Majella	Long 14.11466546<br>Lat 42.10505513	372	318	85.3
82	Majella	Long 14.08376000<br>Lat 42.09094000	352	258	73.3
83	Majella	Long 14.11492474<br>Lat 42.10588115	334	241	72.0
84	Majella	Long 14.08470593<br>Lat 42.08640557	297.5	242	81.2
85	Velino	Long 13.37275469<br>Lat 42.15687756	263	221	83.8

86	Majella	Long 14.08839000<br>Lat 42.09355000	352	296	84.1
87	Majella	Long 14.08938000<br>Lat 42.09633000	432.5	354	81.8
88	Majella	Long 14.09599206<br>Lat 42.02822347	267	208	77.7
89	Gran Sasso	Long 13.70125398<br>Lat 42.44349301	165	123	74.2
90	Velino	Long 13.37348677<br>Lat 42.16016755	139.5	131	93.5
91	Majella	Long 14.08068152<br>Lat 42.08777983	260.5	257	98.7
92	Majella	Long 14.09940841<br>Lat 42.02812150	417	384	92.1

The species cover in the original plots expressed on Braun-Blanquet cover scale were transformed on % values as follows +: 0.5 %; 1: 3%; 2: 15%; 3: 37.5%; 4: 62.5%; 5: 87.5%

Table S2

Sensitivity analysis obtained by calculating the correlation among CWM_t and FD_t values, considering the overall cover (cover 100%) and the values that CWM_t and FD_t assumed using gradually reduced species cover (step of 5%). Procedure performed according with Májeková et al. (2016).

		Correlation among CWM_t and FD_t *calculated with different plant cover values*					
		FD_{PMH}	CWM_{PMH}	FD_{SLA}	CWM_{SLA}	FD_{LDMC}	CWM_{LDMC}
COVER	100%	1	1	1	1	1	1
	95%	0.992818	0.991816	0.966	0.989088	0.971795	0.993249
	90%	0.975001	0.98132	0.961716	0.986098	0.964552	0.986514
	85%	0.941972	0.945529	0.684211	0.974412	0.827117	0.913116
	80%	0.884355	0.905768	0.637496	0.957277	0.792835	0.877378
	75%	0.8392	0.900451	0.613375	0.950803	0.782158	0.870273

Table S3

List of the dominant species (**species**, asterisks indicate the Apennine endemic taxa), along with their taxonomic family (**Family**), Growth form (**GF** - Raunkiaer 1934, Pignatti 2019; CH FRUT: fruticose chamaephyte, CH PULV: pulvinate chamaephyte, CH REPT: reptant chamaephyte ,CH SUFFR: suffruticose chamaephyte, H SCAP: scapose hemicryptophyte , H ROS: hemicryptophyte with rosette , H CAESP: caespitose hemicryptophyte), number of plots in which the species occurs (**N pl**), mean measured traits (**SLA:** specific leaf area, **LDMC:** leaf dry matter content, **PMH:** maximum plant height), percentage of plots in which the species occurs into the alpine *Silene acaulis* community (**% pl alpine**) and **into the** subalpine *Sesleria juncifolia* community (**% pl subalpine)**

Species	Family	GF	N pl	SLA (mm²/mg)	LDMC (mg/g)	PMH (cm) (S.D)	% pl alpine	% pl subalpine
Alyssum cuneifolium subsp. *cuneifolium*	Brassicaceae	CH SUFFR	8	12.38	207.7	2.9 (0.85)	0.09	-
Androsace villosa subsp.*villosa*	Primulaceae	CH REPT	35	15.83	237.03	1.79 (0.35)	-	0.27
**Androsace vitaliana* subsp. *praetutiana*	Primulaceae	CH SUFFR	4	17.34	224.89	0.55 (0.52)	-	0.22
Anthyllis montana	Fabaceae	CH SUFFR	38	8.94	305.59	5.46 (1.25)	0.15	0.03
Anthyllis vulneraria subsp. *pulchella*	Fabaceae	H SCAP	65	11.65	207.89	4.49 (1.46)	0.11	-
Arenaria grandiflora subsp. *grandiflora*	Caryophyllaceae	CH SUFFR	18	14.46	293.68	2.2 (0.49)	0.76	0.51
**Armeria gracilis* subsp. *majellensis*	Plumbaginaceae	H ROS	33	19.46	207.15	8.60 (3.32)	0.38	0.38
Aster alpinus subsp. *alpinus*	Asteraceae	H SCAP	19	12.12	301.42	7.13 (3.94)	0.24	0.41
**Brachypodium genuense*	Poaceae	H CAESP	9	16	501.68	36.12 (6.72)	0.02	0.08
Bromopsis erecta	Poaceae	H CAESP	17	11.34	477.28	32.72 (7.04)	0.04	0.38
Carex humilis	Cyperaceae	H CAESP	32	9.01	395.26	11.66 (3.06)	0.62	0.03
Carex kitaibeliana subsp. *kitaibeliana*	Cyperaceae	H CAESP	68	11.27	360.3	10.96 (2.92)	0.07	-
Carex macrolepis	Cyperaceae	H CAESP	5	9.51	416.35	29.86 (5.12)	0.91	0.57
**Cerastium thomasii*	Caryophyllaceae	CH SUFFR	10	13.78	321.03	2.4 (0.79)	0.64	0.08
**Cerastium tomentosum*	Caryophyllaceae	CH SUFFR	9	12.05	361.73	5.13 (2.50)	0.71	0.7
Cytisus spinescens	Fabaceae	CH SUFFR	6	10.08	309.72	13.33 (4.34)	0.31	0.24
Edraianthus graminifolius subsp. *graminifolius*	Campanulaceae	CH SUFFR	61	13.05	245.61	2.79 (1.49)	0.29	0.16
**Festuca violacea* subsp. *italica*	Poaceae	H CAESP	28	11.42	345.6	6.89 (2.71)	0.09	0.35

**Galium magellense*	Rubiaceae	H SCAP	16	23.2	207.65	2.46 (0.87)	0.13	0.7
Globularia meridionalis	Plantaginaceae	CH REPT	35	8.38	307.25	2.06 (0.59)	0.05	0.32
Helianthemum nummularium subsp. *grandiflorum*	Cistaceae	CH SUFFR	4	14.06	257.63	15.06 (8.06)	0.25	0.22
Helianthemum oelandicum subsp. *alpestre*	Cistaceae	CH SUFFR	71	8.31	353.13	6.99 (1.83)	0.11	0.3
**Helictochloa praetutiana*	Poaceae	H CAESP	26	14.05	444.70	24.29 (2.33)	0.31	0.05
Iberis saxatilis subsp. *saxatilis*	Brassicaceae	CH SUFFR	22	9.36	252.608	3.22 (0.66)	0.24	0.11
Kobresia myosuroides	Cyperaceae	H CAESP	15	13.85	386.797	8.5 (0.66)	0.55	0.43
Leontopodium nivale	Asteraceae	H SCAP	22	13.02	245.939	3.72 (1.81)	0.11	0.03
**Myosotis graui*	Boraginaceae	H SCAP	17	19.24	190.294	2.34 (0.63)	0.16	-
Oxytropis campestris subsp. *campestris*	Fabaceae	H SCAP	17	9.5	280.347	4.4 (0.698	0.13	0.27
**Pedicularis elegans*	Orobanchaceae	H ROS	46	9.53	257.52	4.4 (1.42)	0.29	0.03
Potentilla apennina subsp. *apennina*	Rosaceae	CH SUFFR	7	10.45	405.887	3.8 (1.32)	0.05	0.68
Potentilla crantzii	Rosaceae	H SCAP	17	11.63	364.165	7.46 (2.13)	0.96	0.22
Salix retusa	Salicaceae	CH FRUT	28	12.48	309.169	2.89 (0.84)	0.25	0.7
Sesleria juncifolia subsp. *juncifolia*	Poaceae	H CAESP	61	9.5	405.004	16.78 (5.32)	0.35	0.22
Silene acaulis subsp. *bryoides*	Caryophyllaceae	CH PULV	40	12.68	256.71	1.25 (0.65)	0.45	0.19
Thymus praecox subsp. *polytrichus*	Lamiaceae	CH REPT	27	13.04	329.597	5.33 (1.63)	0.04	0.22
Trinia dalechampii	Apiaceae	H SCAP	32	10.74	225.794	3 (0.82)	-	0.22
**Valeriana saliunca*	Caprifoliaceae	H SCAP	10	14.53	204.142	3.44 (1.57)	0.58	-
**Viola magellensis*	Violaceae	H SCAP	8	16.35	207.729	2.73 (0.76)	0.73	0.76
